# What do windsurfers and kitesurfers in Germany know about surfer's ear and how is it influenced by protective measures?

**DOI:** 10.1017/S0022215123000610

**Published:** 2024-01

**Authors:** F Wegener, M Wegner, N M Weiss

**Affiliations:** 1 Institute of Sport Science, Christian-Albrechts University, Kiel, Germany; 2 Department of Otorhinolaryngology, Head and Neck Surgery, Ruhr University Bochum, St Elisabeth Hospital, Bochum, Germany; 3 Department of Translational Neurosciences, Faculty of Medicine and Health Sciences, Antwerp University, Antwerp, Belgium; 4 International Graduate School of Neuroscience, Ruhr University Bochum, Bochum, Germany

**Keywords:** Quality of life, infectious diseases, external otitis, tinnitus, hearing loss

## Abstract

**Objective:**

This study investigated the frequency of ear canal protection use and looked at its influence on external auditory exostosis severity and knowledge about external auditory exostosis among windsurfers and kitesurfers on the German coast.

**Method:**

This retrospective cross-sectional study interviewed 130 windsurfers and kitesurfers along the German coast on knowledge of external auditory exostosis, exposure time, use of neoprene hoods and earplugs, and otological complaints. Participants underwent bilateral video-otoscopic examination.

**Results:**

Knowledge of external auditory exostosis was ‘good’ or ‘excellent’ in 78 of 130 (60 per cent) individuals and ‘poor’ or non-existent in 52 of 130 (40 per cent) individuals. Knowledge was positively correlated with hours of exposure, otological complaints and frequency of ear canal protection use. A significant negative influence of neoprene hood use on external auditory exostosis severity was shown.

**Conclusion:**

The positive effect of external auditory exostosis knowledge on the frequency of ear canal protection and the reduction of external auditory exostosis risk implies a need for health education on this topic.

## Introduction

External auditory exostosis, also known as ‘surfer's ear’,^1^ is a benign and often bilateral and symmetrical bone growth in the bony part of the external auditory canal. Persistent or recurrent symptoms may result if the exostoses impair the transport and self-cleaning function of the external auditory canal in advanced stages.^2,3^ Depending on the severity, discomfort may be an indication for surgical external auditory exostosis removal.^4,5^ Regular and long-term exposure to water and wind has been reported to be responsible for the development of external auditory exostosis.^1,6–9^ There is evidence that the influence of water increases with decreasing temperature.^10–13^ Individuals with frequent water contact, such as surfers,^7,13–26^ whitewater kayakers^27,28^ and divers,^12,29,30^ have external auditory exostosis prevalence rates ranging from 38 to 90 per cent.^13,26^ Windsurfers and kitesurfers in Germany, who are exposed to wind (approximately 4–8 Beaufort) and to water (approximately 4–18 °C) at the same time, show an external auditory exostosis prevalence of 75 per cent and seem to have a faster development of exostoses than surfers.^9^

Although awareness and knowledge of external auditory exostosis are high among most populations of surfers, awareness and knowledge have been shown to be lower among studied populations who practise open-water swimming, scuba diving and triathlon (Table 1).

**Table 1.** Proportion of water sports athletes with awareness or knowledge of external auditory exostosis

Sports discipline	Country	Participants (*n*)	Proportion of people with awareness (%)	Proportion of people with knowledge* (%)
Surfing	Ireland^32^	207	92	86
	Australia^18^	93	88	
	Japan^24^	373	87	
	UK^31^	375	86	77
	UK^3^	92	78	
	Spain^25^	41	73	
	Ireland^16^	88	65	
Kayaking	Ireland^32^	196	79	62
Open-water swimming		263	59	49
Scuba diving		30	53	37
Triathlon		178	40	30

* ‘Good’ or ‘excellent’ knowledge of external auditory exostosis according to Morris *et al.*^31^

Knowledge about external auditory exostosis prevention methods was confirmed in 55 of 92 (60 per cent) surfers in a study from the UK, 67 of 93 (72 per cent) surfers in a study from Australia and 16 of 23 (70 per cent) surfers in another study from Australia.^3,17,18^

Morris *et al.*^31^ determined that skill level, earplug use and diagnosis of external auditory exostosis had a significant positive effect on knowledge of external auditory exostosis among surfers in the UK, whereas otological complaints and years of exposure were not associated with this knowledge. Similarly, among aquatic athletes in Ireland, Boyle *et al.*^32^ found that years of exposure did not significantly influence knowledge, but knowing someone with external auditory exostosis significantly increased knowledge. Among surfers in the UK who were studied by Reddy *et al.*,^3^ it was found that those with knowledge of the preventability of external auditory exostosis were significantly more likely to use external auditory canal protection than surfers without such knowledge.

Despite the relatively high proportion of surfers with awareness and knowledge of external auditory exostosis, 88 per cent^16^ and 96 per cent^32^ of surfers surveyed in Ireland denied knowledge of the existence of their own external auditory exostosis. Simas *et al.*^33^ also concluded that there was low awareness of external auditory exostosis occurrence after estimating a relatively low external auditory exostosis prevalence of 29 per cent for surfers in New Zealand via an online survey, whereas a physical survey via otoscope in New Zealand showed an external auditory exostosis prevalence of 73 per cent.^23^ Similarly, Nathanson *et al.*^34^ found an external auditory exostosis prevalence of 14 per cent in predominantly American surfers via online survey, while surveys conducted via otoscope found that the prevalence of external auditory exostosis among surfers in the USA ranged from 38 to 74 per cent.^13,22^

### Use of external auditory canal protection

**Figure 1.**
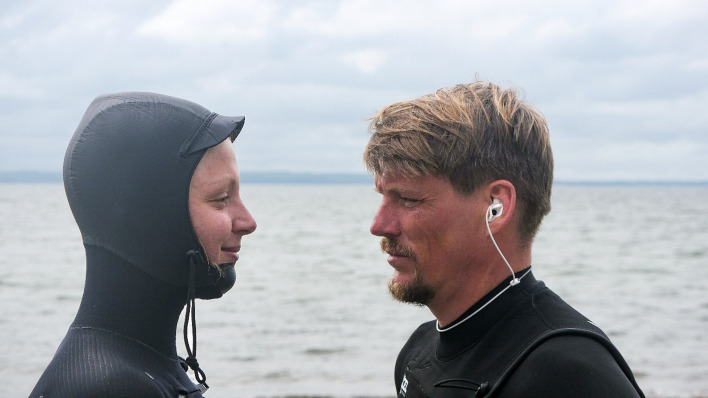
Photograph showing neoprene hood (left) and earplugs (right).

In most cases, the use of external auditory canal protection by water sports athletes refers to the use of a neoprene hood and earplugs (Figure 1). In addition, there is information that ear drops and self-adapted earplugs made of a putty material (e.g., Blu-Tack^®^) are occasionally used.^14,17,18,35,36^

Although neoprene hoods cannot completely prevent water from entering the external auditory canal and are primarily used to protect the head from cold and wind, earplugs are used solely to seal or protect the external auditory canal and eardrum. Cullen^37^ found no water in the external auditory canal in 56 of 60 (93 per cent) swimmers who used earplugs. Requirements for earplugs include a good fit to the external auditory canal, comfortable wearability, affordability, and low impact on hearing and balance.^38^ The individual anatomy of the external auditory canal can affect the fit of the earplug and thus the comfort and seal, which is why customised silicone products are also used.^39^ In a study by Laitakari *et al.*,^40^ earplugs made of mouldable plastic materials (e.g. silicone wax) and a polymeric foam plug treated with petroleum jelly provided a high level of protection against water ingress. In contrast, a cotton plug treated with petroleum jelly and earplugs made of hard acrylic allowed water to enter the external auditory canal.^40^

The use of earplugs and neoprene hoods is relatively low in the populations studied. However, there are large regional differences (Table 2).

**Table 2.** Proportion of individuals who use earplugs or a neoprene hood when surfing

Country	Total (*n*)	Earplugs (%)	Neoprene hood (%)
USA^20^	21	19	–
USA^34^	1348	17	–
New Zealand^23^	92	8	–
Australia^17^	23	30	–
Australia^35^	85	9	6
Japan^24^	373	24	–
Spain^25^	41	0	–
UK^14^	207	18	19
UK^15^	105	22	–
UK^31^	375	40	–
Ireland^32^	207	50	67
Ireland^16^	119	37	87
France^26^	135	40	64

A positive attitude towards the use of earplugs was reported by 70 per cent^31^ and 56 per cent^32^ of surfers surveyed in the UK. According to Nakanishi *et al.*,^24^ earplugs in surfing are used more often by professionals than by amateurs. In contrast, Simas *et al.*^18^ confirmed the use of hearing protection equipment in 17 out of 93 (18 per cent) amateurs and only 1 out of 20 (5 per cent) professional surfers.^18^ The reasons for using external auditory canal protection are usually associated with advanced external auditory exostosis severity and otological complaints.^15,21,26,27^

In water sports, the use of earplugs is advocated or recommended by numerous studies to prevent external auditory exostosis.^1,15,17,19,21,24,26,31,41^ Occasionally, the use of a neoprene hood is also recommended to prevent external auditory exostosis development.^3,42,43^ Case reports, expert opinions and studies exist on the effectiveness of earplugs and neoprene hoods. For example, van Gilse^6^ diagnosed external auditory exostosis in 9 out of 10 professional swim instructors. Only the external auditory exostosis-free individual reported the use of external auditory canal protection. For individuals active in high diving and swimming, Meyer^44^ reported that the frequency of external auditory exostosis was reduced if a swim cap was used.

Harrison^10^ also found that external auditory exostosis was absent in 87 swimming athletes who used external auditory canal protection. Dettmann and Reuter^45^ confirmed that most swimming athletes with external auditory exostosis only occasionally or never wore external auditory canal protection. As described by DiBartolomeo^42^, a surfer successfully protected the right external auditory canal from external auditory exostosis development with an earplug after a ruptured eardrum and subsequent surgery, whereas the unprotected external auditory canal showed multiple external auditory exostosis of moderate severity after 10 years. Timofeev *et al.*^21^ determined a five-fold longer recurrence-free time for UK water athletes who had already undergone surgical external auditory exostosis removal if they used earplugs or a combination of earplugs and a neoprene hood than if they were active with no external auditory canal protection or with only a neoprene hood.

Moore *et al.*^27^ showed that long-term earplug use was significantly associated with lower external auditory exostosis severity in kayakers and concluded that earplug use may decrease the progression of external auditory exostosis. With the use of a neoprene hood or earplugs, the chance of higher severity also decreases significantly among surfers in the UK. However, no additional benefit of the simultaneous use of a neoprene hood and earplugs was observed.^14^ Although time spent in the water without protection or with only a neoprene hood positively correlated with external auditory exostosis severity in surfers in France, such a relationship was not shown between time spent in the water with earplugs and external auditory exostosis severity.^26^ In an additional 926 participants who engaged in various ‘cold water sports’, the use of earplugs was found to have a significant reducing effect on otological discomfort, whereas the use of a neoprene hood had no protective effect.^32^ In contrast, Deleyiannis *et al.*^20^ found no significant difference in external auditory exostosis severity between surfers who used a neoprene hood or earplugs for more or less than 50 per cent of their exposure time. Chaplin and Stewart^23^ also determined no significant difference in external auditory exostosis severity between surfers and lifeguards who used earplugs and those who did not. Similarly, Nakanishi *et al.* did not find a protective effect of earplugs.^24^ This effect was explained by the observation that the earplugs were used only after complaints of existing external auditory exostosis occurred.^24^ Similarly, Lennon *et al.*^16^ and Simas *et al.*^35^ found no significant relationship between the use of earplugs and external auditory exostosis severity in surfers.

In summary, the use of external auditory canal protection and the knowledge of external auditory exostosis varies considerably among water sports participants. Often, the use of external auditory canal protection and the knowledge of external auditory exostosis increases with increasing complaints. The effectiveness of external auditory canal protection appears to be higher with earplugs than with a neoprene hood.

Therefore, the aim of this study was to determine the level of knowledge about external auditory exostosis among windsurfers and kitesurfers on the German coast. In addition, the frequency of use of external auditory canal protection and its influence on external auditory exostosis severity was investigated. In particular, this study aimed to re-evaluate the utility of neoprene hoods, whose benefit was previously described as less than that of earplugs, among German windsurfers and kitesurfers because neoprene hoods are used particularly frequently in this population.

## Materials and methods

In a retrospective cross-sectional study, German non-professional windsurfers and kitesurfers were recruited between September 2020 and November 2020 along the North and Baltic Sea coasts. Participants were eligible for inclusion if they reported a frequency of windsurfing and/or kitesurfing of at least 75 per cent at the German coast and a frequency of other outdoor water sports of less than 25 per cent. They were excluded if the external auditory canal could not be clearly examined or if they had a history of surgical removal of external auditory exostosis. The study was approved by the local ethics committee of the Institute of Sport Science at the University of Kiel.

External auditory exostosis awareness was assessed using a yes-or-no questionnaire, as previously reported in other studies.^3,24,31,32^ Individuals with awareness of the topic were asked to rate 10 statements in German that were modified from Morris *et al.*^31^ to determine their knowledge of external auditory exostosis.

The surveyed windsurfers and kitesurfers indicated their exposure time in years and, for each season, their exposure frequency in days per week and hours per day. The exposure time in hours was calculated from these parameters. In addition, changes in activity (e.g. because of injury) were considered in the quantitative interview.

The frequency of use of hearing protection equipment in the form of earplugs or a neoprene hood was surveyed for each season and classified as follows: 0 per cent, no use; 25 per cent, low use; 50 per cent, partial use; 75 per cent, frequent use; and 100 per cent, constant use. Additionally, the time when they began to use ear protection was recorded. With this information, the ratio of exposure time with and without the use of hearing protection equipment was determined.

Otological complaints (e.g. water retention, itching, hearing loss, inflammation, tinnitus) were assessed for the past 12 months. As in the study by Alexander *et al.*,^14^ the degree of discomfort was differentiated into medically minor or requiring medical treatment.

The external auditory canal was examined using a portable digital video otoscope (XION Medical GmbH, Berlin, Germany). The otoscopic images (software DiVAS Mini version 3.2 by XION) were evaluated by one senior physician who specialised in otology (NW). External auditory exostosis severity was described using a four-point scale based on similar previous studies: 0, no visible obstruction; 1, mild (less than one-third obstructed); 2, moderate (one-third to two-thirds obstructed); and 3, severe (more than two-thirds obstructed).^4,7,9,18,24^

### Statistical analysis

All statistical tests were selected before data collection. Statistical analyses were performed using Microsoft Excel (version 21.01, Microsoft Corporation, Redmond, USA) and SPSS (version 27, IBM SPSS Statistics, New York, USA). If not otherwise specified, data are presented as the mean with standard deviation (SD) and absolute numbers (*n*) with percentages. The significance level was set to *p* < 0.05. Correlations were performed for categorical variables using Spearman rank-order correlation (*r_s_*). For continuous variables, Pearson correlation (*r_p_*) was used. The effect of frequency of use of external auditory canal protection on external auditory exostosis severity was examined using multiple ordinal regression analysis after elimination of one outlier. Since statistical test procedures are performed under the assumption that the measured values are independent from each other,^46^ only one measured value of external auditory exostosis severity per examined person was included in the analysis. In cases of asymmetric obstruction, the more severely affected external auditory canal was used for analysis, as applied in previous studies.^7,13,14,24,27^

## Results

Twenty-two females (17 per cent) and 108 (83 per cent) males were included in the study, with a mean age of 35.5 years (SD = 10.8 years). A total of 43 (33 per cent) individuals were kitesurfers only, 55 (42 per cent) individuals were windsurfers only and 32 (25 per cent) individuals were kitesurfers with a history of windsurfing.

Of all the participants, 89 (68 per cent) were predominantly active in the Baltic Sea, 16 (12 per cent) in the North Sea and 25 (19 per cent) in equal proportions in the North and Baltic Seas. The exposure time by season was as follows: 27 per cent for spring, 33 per cent for summer, 29 per cent for autumn and 11 per cent for winter.

### Knowledge about external auditory exostosis

A total of 105 (81 per cent) windsurfers and kitesurfers surveyed confirmed that they were aware of the topic of ‘surfer's ear’. Among these participants, knowledge of the topic was rated as ‘excellent’ by 10 (10 per cent) participants, ‘good’ by 68 (65 per cent) participants and ‘poor’ by 27 (26 per cent) participants. The 10 statements on the topic ‘surfer's ear’ were rated mostly correct (71 per cent; Table 3). Statements that were most frequently rated incorrectly concerned the following topics: ‘causal treatment only by surgery’ (statement 1), ‘bone growth’ (statement 8) and ‘long-term period of formation’ (statement 10).

**Table 3.** Summary of responses on windsurfers’ and kitesurfers’ knowledge about external auditory exostosis (*n* = 105)

Number	Statement	Incorrect		Correct	
		Value (*n*)	Value (%)	Value (*n*)	Value (%)
1	Surgery is the only cure for external auditory exostosis	60	57	45	43
2	Wind chill contributes to external auditory exostosis	25	24	80	76
3	All surfers are at risk for external auditory exostosis	29	28	76	72
4	External auditory exostosis can completely close up the ear canal	37	35	68	65
5	External auditory exostosis is due to cold water exposure	20	19	85	81
6	You cannot see external auditory exostosis by looking in the mirror	7	7	98	93
7	External auditory exostosis can be prevented	12	11	93	89
8	External auditory exostosis is due to bone growth in the ear canal	39	37	66	63
9	External auditory exostosis features ear infections & hearing loss	28	27	77	74
10	Only long-term surfers get external auditory exostosis	45	43	60	57
	Mean	30.2	29	74.8	71

In summary, knowledge of the topic was rated as ‘good’ or ‘excellent’ for 78 (60 per cent) participants in the total sample and ‘poor’ or no knowledge was identified in 52 (40 per cent) participants. Knowledge about external auditory exostosis is positively correlated with hours of exposure to windsurfing and kitesurfing (*r_p_* = 0.248; *p* ≤ 0.01), otological discomfort level (*r_s_* = 0.274; *p* ≤ 0.01) and use of external auditory canal protection (*r_p_* = 0.241; *p* ≤ 0.01). These three correlations are weak effects according to Cohen.^47^

### Use of external auditory canal protection

**Figure 2.**
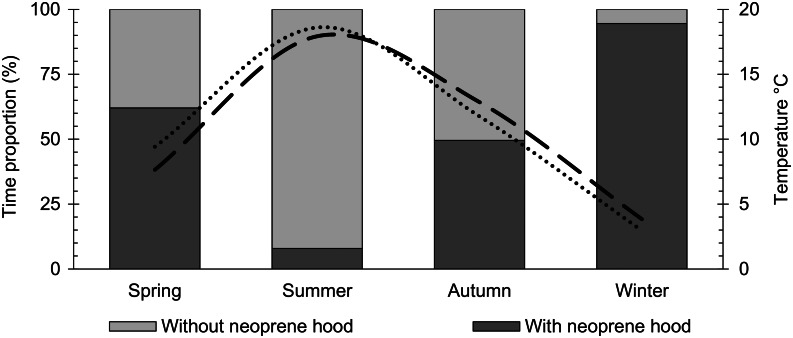
Graph showing the percentage of time a neoprene hood is used for each season.

Neoprene hoods were used by 124 (95 per cent) windsurfers and kitesurfers during an average of 44 per cent (SD = 18.7) of the exposure time. Earplugs were used by 28 (22 per cent) individuals during an average of 11 per cent (SD = 10.2) of the exposure time. In response to otological discomfort, 19 of 28 (68 per cent) participants used earplugs, and 7 of 124 (6 per cent) participants used a neoprene hood. Because of the infrequent use of earplugs, the following analyses refer only to the use of the neoprene hood. Although 17 (13 per cent) individuals used a neoprene hood in summer, 114 (88 per cent) individuals used it in autumn and 122 (94 per cent) individuals used it in spring. Of 107 people active in winter, 105 (98 per cent) people used a neoprene hood. The percentage of time a neoprene hood is used is shown for each season in Figure 2.

The regression analysis shows a significant negative influence of the frequency of neoprene hood use on external auditory exostosis severity. For each additional percentage of neoprene hood use, the chance of high external auditory exostosis severity decreases by a factor of 0.036 (0.965−1) or 4 per cent (Table 4).

**Table 4.** Regression analysis of the influence of neoprene hood use and hours of exposure on external auditory exostosis severity

Predictor	β	SE	Wald	df	*P-*value	OR	95 % CI	
Neoprene hood use	−0.036	0.009	15.678	1	≤0.001	0.965	−0.055	−0.018
Exposure hours								
– <600	−5.622	0.747	56.705	1	≤0.001	0.004	−7.086	−4.159
– ≥ 600 to < 1400	−4.474	0.685	42.626	1	≤0.001	0.011	−5.817	−3.131
– ≥ 1400 to < 2200	−2.608	0.619	17.726	1	≤0.001	0.074	−3.822	−1.394
– ≥ 2200 to < 3600	−2.324	0.622	13.952	1	≤0.001	0.098	−3.544	−1.105
– ≥ 3600	(Reference)							

N = 129, −2 log likelihood = 250.270, chi squared = 91.632, df = 5, p ≤ .001. R^2^: Cox and Snell = 0.509, Nagelkerke = 0.544, McFadden = 0.259. SE = standard error; df = degrees of freedom; OR = odds ratio; CI = confidence intervals

As an example, the influence of neoprene hood use will be illustrated by a selected participant (number 1 in the dataset). This participant reported an exposure time of equal to or more than 3600 hours with a neoprene hood use rate of 56 per cent. Based on the regression model, this individual's expected probabilities for external auditory exostosis severity are shown in Table 5. Additionally, listed in this overview are the probabilities of the external auditory exostosis severities assuming 90 per cent neoprene hood use for this individual. As the neoprene hood usage increases by approximately 30 per cent, the expected external auditory exostosis severity decreases from ‘severe’ to ‘moderate’.

**Table 5.** Probabilities of external auditory exostosis severity with varying neoprene hood use for individuals with equal or more than 3600 hours of exposure

External auditory exostosis severity	Probability at 56% neoprene hood use (%)	Probability at 90% neoprene hood use (%)	Difference (%)
Normal	1	2	+1
Mild	5	15	+10
Moderate	29	45	+17
Severe	65	38	–28

## Discussion

This study evaluated the association of external auditory exostosis in windsurfers and kitesurfers with knowledge of this topic and the use of hearing protection measures. The reliability analysis of the knowledge assessment from Morris *et al.*^31^ shows good internal consistency, with a Cronbach's alpha value of 0.82.^48^ With a discriminatory power of equal to or more than 0.4 each, the items differentiate similarly to the overall construct and are therefore able to measure it effectively.^49,50^

The proportion of windsurfers and kitesurfers who are aware of external auditory exostosis can be considered high (81 per cent), which is similar to the results in surfers (65 per cent to 92 per cent).^3,16,18,24,25,31,32^ Overall, 60 per cent of windsurfers and kitesurfers have ‘good’ or ‘excellent’ knowledge and 40 per cent have ‘poor’ or no knowledge about external auditory exostosis, showing a lower rate of good or excellent knowledge than 375 surfers in the UK (77 per cent)^31^ and 207 surfers in Ireland (86 per cent).^32^ Because of a relatively large number of studies and spread of information about external auditory exostosis via social media, the topic is more commonly discussed in relation to surfing than in relation to windsurfing and kitesurfing. This may explain the higher knowledge of surfers in the UK and Ireland. Among windsurfers and kitesurfers in this study, knowledge about external auditory exostosis was shown to be significantly positively related to exposure time and otological discomfort level. This may suggest that surfers are more concerned with the topic after complaints have already arisen. This is consistent with the results of Wille *et al.*,^36^ who demonstrated a significant positive association of knowledge about external auditory exostosis and exposure time among 81 whitewater kayakers in the USA but is contrary to the results of Morris *et al.*^31^ Overall, there was a high level of interest in information about external auditory exostosis among the windsurfers and kitesurfers in the study. This is similar to results found in the UK, where 84 per cent of surfers surveyed suggested that they would benefit from more health advice on the topic.^31^

### Use of external auditory canal protection

Temporary use of the neoprene hood was reported by 124 (95 per cent) windsurfers and kitesurfers. Because of the low water and air temperatures on the German coasts in autumn, winter and spring, the neoprene hood is an essential part of thermal insulation equipment. In climatically similar regions, the percentage of surfers using a neoprene hood is also high, with 87 per cent^16^ and 67 per cent^32^ in Ireland and 64 per cent in France.^26^ In contrast, the relatively low neoprene hood use of only 19 per cent of the surfers surveyed by Alexander *et al.*^14^ in the UK is remarkable because the water temperatures of 4 to 18 °C are also relatively low and would suggest greater use. The low neoprene hood use of only 6 per cent of surfers surveyed in Australia is plausible because the water temperature is 19 to 28 °C there.^35^

The temporary use of earplugs is confirmed by 22 per cent of windsurfers and kitesurfers. Among surfers in climatically similar regions, the use of earplugs is heterogeneous. As an example, 18 per cent^14^ and 22 per cent^15^ of surfers in the UK and 37 to 50 per cent of surfers in Ireland, France and the UK confirmed the use of earplugs.^16,26,31,32^ According to Kristen *et al.*,^51^ windsurfers rarely use earplugs because they are considered responsible for a reduced ability to assess wind direction and strength. However, the use of earplugs may also be low among windsurfers and kitesurfers because they are associated with uncomfortable wearing and limitations in communication, balance and performance. Surfers cite these reasons primarily for not using earplugs.^8,18,31^

Because the feeling of freedom and an opposition to regulation are central aspects of motivation for windsurfers and kitesurfers,^52,53^ the use of earplugs could also be perceived as a form of compulsion, obligation or paternalism and might consequently be rejected. This is also supported by the low use of helmets or impact vests. These protective measures were worn by only 3 of 18 (17 per cent) windsurfers and 7 of 26 (27 per cent) kitesurfers in the Netherlands.^54^ The use of earplugs is attributable to otological discomfort in the majority of windsurfers and kitesurfers (68 per cent). Similarly, such a pattern has been identified in surfers and whitewater kayakers.^15,21,26,27^ Because complaints usually occur after a number of years, the average percentage of time spent in the water with earplugs was only 11 per cent in the present study. Based on these weak data, an analysis of the efficacy of earplugs was not possible. In contrast, Lambert *et al.*^26^ reported the use of earplugs during 64 per cent of the exposure time in 135 surfers in France and an associated significant protective effect against external auditory exostosis development. Other studies confirm the protective effect of earplugs in terms of protection against water^37^ and the development of external auditory exostosis.^14,21,26,27^

In the present study, there was a significant positive association between knowledge about external auditory exostosis and the frequency of use of earplugs. Consistent with this, 56 and 80 per cent of surfers in the UK who do not use earplugs confirmed that they would consider using them in the future if they knew more about external auditory exostosis.^3,31^ Promisingly, 60 of 80 (75 per cent) whitewater kayakers confirmed a greater motivation to use ear protection after being informed about external auditory exostosis.^36^

Comparable to the results of Alexander *et al.*^14^ with regard to surfers, increasing use of the neoprene hood significantly reduces the risk of higher external auditory exostosis severity in windsurfers and kitesurfers. Timofeev *et al.*^21^ confirmed the protective effect of a neoprene hood only in combination with earplugs, and Lambert *et al.*^26^ and Deleyiannis *et al.*^20^ reported no protective effect of neoprene hood use on external auditory exostosis severity in surfers. It is conceivable that a neoprene hood has a stronger protective effect in windsurfing and kitesurfing because it is not only a barrier to water but primarily protects against wind, which is not present at a similar strength in surfing. Notably, the neoprene hood was used by the surfers studied by Lambert *et al.*^26^ only during 25 per cent of the time spent in the water. It is possible that a protective effect may only occur after more regular use and is statistically more difficult to identify from this relatively low use.

However, the results of the present study also indicate the limits of the protective effect of the neoprene hood. Thus, with continuous use, external auditory exostosis development can be decreased but probably not completely prevented. This can be explained by the fact that the external auditory canal is not fully protected against water and wind by the neoprene hoods available to date. Earplugs could be a useful addition in this context.^14,21,26,27,37^ Although other studies do not provide significant results on the effectiveness of earplugs,^16,20,23,24,35^ it is believed that they can provide a functional barrier to water and wind for windsurfing and kitesurfing. Especially on days with temperatures too high for the use of a neoprene hood, earplugs could be used as an alternative.

### Limitations

According to the inclusion criteria, the present study is limited to windsurfers and kitesurfers on the German coast and cannot be generalised to all windsurfers and kitesurfers worldwide. This is especially the case for individuals from regions with entirely different water and air temperatures.

For participants with a long history of participation, it was difficult to assess total exposure time. However, the survey was very detailed and episodes with changing frequency of activities were considered.

This study did not assess the quality of external auditory canal protection. Neoprene hoods that form a fixed unit with the wetsuit offer better protection because there is almost no possibility of water entering through the neck area compared with neoprene hoods worn separately. Similarly, how well the neoprene hood fits the face area may be critical in acting as a barrier to water and wind. Earplugs may also provide better protection of the external auditory canal if they are custom fitted and made of silicone.^40^

- Compared with populations of surfers, data show that windsurfers and kitesurfers have less knowledge about external auditory exostosis, also known as ‘surfer's ear’
- Knowledge of external auditory exostosis in windsurfers and kitesurfers is also often obtained in the context of otological complaints, which are usually associated with long-term activity
- Windsurfers and kitesurfers used neoprene hoods more frequently than earplugs, and the use of ear canal protection is associated with increased knowledge about external auditory exostosis
- A neoprene hood provides protection against external auditory exostosis formation in windsurfers and kitesurfers
- The spread of information about external auditory exostosis among windsurfers and kitesurfers should be encouraged

## Conclusion

Knowledge about external auditory exostosis is shown to be ‘good’ or ‘excellent’ in more than half of the respondents. However, knowledge is often acquired in the context of otological complaints, which usually arise after many years of activity. The use of protective measures, which increases with knowledge about external auditory exostosis, should encourage the spread of information about external auditory exostosis among windsurfers and kitesurfers. Thus, a contribution can be made to reduce external auditory exostosis prevalence in windsurfers and kitesurfers.

A protective effect of neoprene hoods on external auditory exostosis development against water and wind may be concluded. However, this barrier does not yet achieve complete protection and is probably unable to completely prevent external auditory exostosis development. In this context, earplugs could be used as a complementary measure.
